# Effects of cancer on stroke recurrence and mortality: A single-center retrospective cohort study

**DOI:** 10.1016/j.ensci.2023.100474

**Published:** 2023-07-17

**Authors:** Kang-Po Lee, Hui-Chi Huang, Jui-Yao Tsai, Li-Chi Hsu

**Affiliations:** a Department of Neurology, E-Da Hospital, Kaohsiung, Taiwan; b School of Medicine for International Students, College of Medicine, I-Shou University, Kaohsiung, Taiwan; c Department of Nursing, Taipei Veterans General Hospital, Taipei, Taiwan; d Division of Cerebrovascular Diseases, Neurological Institute, Taipei Veterans General Hospital, Taipei, Taiwan; e School of Medicine, National Yang Ming Chiao Tung University College of Medicine, Taipei, Taiwan

**Keywords:** cancer, Ischemic stroke, Prognosis, Recurrence, Mortality

## Abstract

**Background:**

Whether patients with stroke and cancer have specific characteristics remains controversial. In addition, research regarding the effects of individual cancer types on stroke outcomes remains scarce. This study investigated the mortality and stroke recurrence rates in patients with stroke and concomitant cancer and evaluated outcome predictors.

**Methods:**

This study retrospectively enrolled 2610 patients in the Taipei Veterans General Hospital Stroke Registry registered from January 2019 to December 2020. A total of 1868 patients were included after excluding those without acute ischemic stroke or hospitalization. The patients were then categorized into the following diagnostic groups: cancer-associated stroke (CAS), stroke and inactive cancer, and stroke without cancer. The discharge mortality rate, 1-year mortality rate, and 1-year stroke recurrence rate were compared. Multiple clinical characteristics and comorbidities—age, sex, stroke severity, coagulopathy, common vascular risk factors, and acute stroke treatment—were also assessed.

**Results:**

A total of 302 (16.2%) patients had concomitant cancer; 39 (2.1%) patients were classified as having CAS and 263 (14.1%) as having stroke with inactive cancer. The baseline characteristics, stroke severity, and type of acute reperfusion therapy were similar among the three groups. However, the stroke recurrence and mortality rates were much higher in the patients with CAS in both short-term and long-term follow-up. The 30-day and 1-year mortality rates for the CAS, inactive cancer, and no cancer groups were 17.9%, 12.5%, and 4.7%, (*p* < 0.001) and 51.3%, 33.8%, and 12.4%, (*p <* 0.001) respectively.

**Conclusion:**

Patients with stroke and active cancer had similar stroke severity. However, the 1-year mortality and stroke recurrence rates were higher in these patients than in patients with inactive cancer or the control group.

## Highlights

• Stroke severity is similar in patients with and without stroke.
• Cancer-associated stroke is associate with the highest recurrence rate and 1-year mortality.
• In patient with cancer-associated stroke, coagulopathy, and high CRP level lead to worse outcomes.
• Hypertension, hyperlipidemia, and overweight are associated with better outcome in stroke patients with inactive cancer.

## 1 Introduction

Stroke is the second‑leading cause of death and the third‑leading cause of disability worldwide [[1], [2], [3]]. Cancer has long been the second‑leading cause of death following cardiovascular disease [4]. Thus, neurologists may encounter patients with both cancer and stroke [5], and an estimated 7%–16% of patients with stroke have concomitant cancer [6]. Stroke etiologies include large artery atherosclerosis (LAA), small vessel occlusion (SVO), cardiac embolism (CE), and other determined etiologies and undetermined etiologies, including cryptogenic stroke [7]. Cancer-associated stroke (CAS) may involve not only these conventional etiologies but also cancer-related factors such as coagulopathy, tumor embolism, or treatment-related factors [8,9]. Adenocarcinoma may account for up to 70% of cancer-related stroke cases [10]. The most common concomitant cancers in patients with stroke are solid tumors of the lung, gastrointestinal tract, and breast [11]. However, whether active cancer in patients with ischemic stroke affects the pathobiology or mechanism of the stroke or patient outcomes remains unclear. Furthermore, the findings regarding initial stroke severity in patients with cancer are controversial; some studies have suggested that patients with cancer have more severe stroke [12], whereas other studies have reported the opposite finding [13]. Scholars have suggested that compared with patients without cancer, patients with stroke and a history of cancer have a higher risk of short-term recurrent stroke and short-term cardiovascular mortality [12,14]. However, few studies have investigated long-term mortality and recurrence rates and the results remain uncertain. Moreover, differences may exist between people of European descent, Asians, and Africans. Data on the Han Chinese population are particularly limited.

Therefore, this study retrospectively analyzed the outcomes of patients with stroke with or without concomitant cancer by comparing in-hospital mortality, 1-year mortality, and 1-year stroke recurrence rates as well as functional independence. In addition, this study investigated outcomes by cancer type.

## 2 Material and methods

### 2.1 Design

This retrospective study evaluated the association and prognosis of CAS by using data from the Taipei Veterans General Hospital Stroke Registry (TVGHSR). The TVGHSR is a prospectively collected stroke registry comprising patients with ischemic and hemorrhagic strokes who were hospitalized in the neurology ward of Taipei Veterans General Hospital (TVGH). The TVGHSR data enabled us to analyze the etiologies, risk factors, demographic characteristics, and outcomes of patients with stroke. The study was approved by the Research Ethics Committee of TVGH (VGHTPE: 2021–02-018A); the need for signed informed consent was waived due to the retrospective and anonymous nature of the study.

### 2.2 Population

**Fig. 1:**
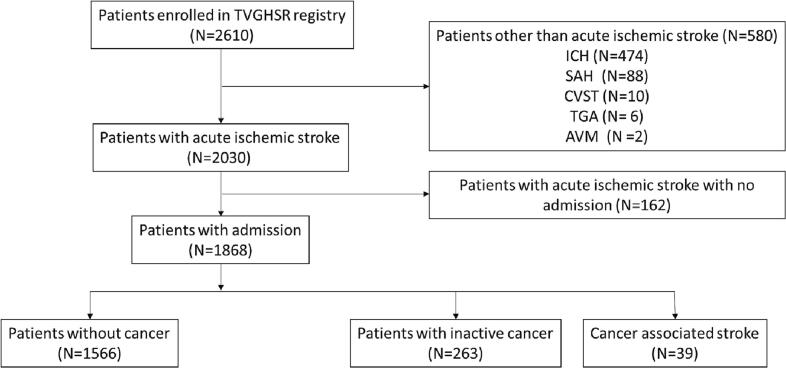
Study inclusion flowchart.

A total of 2610 patients were registered in the TVGHSR between January 2019 and December 2020. In our study, we excluded patients with intracranial hemorrhage (*n* = 474), subarachnoid hemorrhage (*n* = 88), cerebral venous sinus thrombosis (*n* = 10), transient global amnesia (*n* = 6), or intracranial arteriovenous malformation (*n* = 2). Patients with suspected acute ischemic stroke who were not hospitalized (*n* = 162) were also excluded. A total of 1868 patients were included in the final analysis (Fig. 1). The patients had undergone routine examination in accordance with the guidelines of the Taiwan Stroke Society [15]. Those who met either of the following descriptions were classified as having CAS: 1) diagnosis of an embolic stroke of undetermined source with concomitant cancer; or 2) recently (<6 months) received a diagnosis of cancer or was receiving anticancer therapy during admission for acute ischemic stroke. The patients were classified as having stroke with inactive cancer if the etiology of their stroke was LAA, SVO, CE, or another determined cause and they had a concurrent medical history of cancer. The remaining patients were classified as patients with stroke without cancer (i.e., control group). The statistical analysis was performed using demographic data, laboratory findings, and established vascular risk factors extracted from the TVGHSR databank. Body mass index (BMI) was calculated as kilograms divided by the square of the height in meters and classified as indicating underweight, normal weight, and overweight if <18.5, between 18.5 and 23.9, and >24.0 kg/m^2^, respectively. Arterial hypertension (HTN) was defined as systolic blood pressure of ≥140 mmHg or diastolic blood pressure of ≥90 mmHg on two occasions [16] or as the current use of antihypertensive medication. Diabetes mellitus (DM) was diagnosed through a fasting plasma glucose level of >126 mg/dL, hemoglobin A1C (HbA1C)level ≥ 6.5% [17], or current treatment with an oral hyperglycemic agent. Dyslipidemia (DL) was defined as a fasting cholesterol level of >200 mg/dL or the use of a lipid-lowering agent. High levels of low-density lipoprotein–cholesterol (LDL–C) and triglycerides were defined as >100 mg/dL and > 150 mg/dL, respectively [18]. Atrial fibrillation (Afib) was recorded if patients had a medical history of Afib or if it was detected through electrocardiogram (EKG) or a 24-h Holter EKG conducted during hospitalization. Chronic kidney disease (CKD) was defined as an estimated glomerular filtration rate of ≤60 mL/min/1.73 m^2^. End-stage renal disease (ESRD) was defined as receiving regular hemodialysis or peritoneal dialysis. Anemia was defined as a hemoglobin level < 12 g/dL in women and < 13 g/dL in men. The study also recorded patients' medical history, including stroke. The initial stroke's severity was assessed using the National Institute of Health Stroke Scale (NIHSS). The Modified Rankin Scale (mRS; score of 0–6, where 0 indicates no symptoms at all and 6 indicates death) was used for measurement of functional outcomes. After discharge, the patients received follow-up care at TVGH at 3-month intervals for 1 year. Those who received follow-up care in other hospitals were interviewed by telephone at 1, 3, 6, and 12 months after hospitalization for stroke by two experienced stroke case managers from the TVGH stroke center, who recorded their mRS score, time of recurrent stroke, and mortality.

### 2.3 Measurements and statistical analysis

Patient demographic characteristics were obtained from the TVGHSR. For univariate analyses, the chi-square test was performed for categorical variables; independent *t*-tests were used for ordinal and linear variables; and the Mann–Whitney *U* test was employed for nonnormally distributed variables. Statistical significance was indicated at *p* ≤ 0.05. Multivariate analyses were adopted with the Cox proportional-hazards regression model. Kaplan–Meier analyses with the log-rank test were used to analyze the relationship between malignancy and survival rate in patients with stroke. We employed univariate and multivariate logistic regressions to evaluate the associations of parameters with the risk of death after ischemic stroke. All statistical analyses were performed using SPSS Statistics Version 17 (Chicago, IL, USA).

## 3 Results

### 3.1 Patient characteristics

Among the 1868 patients eligible for inclusion in the study, 302 (16.2%) had received a cancer diagnosis previously or during hospitalization. Of these 302 patients, 39 (2.1%) were classified as having CAS and 263 (14.1%) as having stroke with inactive cancer. The CAS group were slightly younger than the inactive cancer group and control group, with the mean ages being 68.3, 73.4, and 72.3 years, respectively (*p* = 0.098). With regard to conventional vascular comorbidity, significantly fewer patients with CAS than those with inactive cancer and the control patients had HTN (66.7%, 85.2%, and 90.9%, respectively; *p* = 0.001), DL (46.2%, 66.5%, and 75.1%; *p* < 0.001), ESRD (2.6%, 1.5%, and 4.7%; *p* = 0.006), and Afib (7.7%, 22.1%, and 19.1%; *p* = 0.019). However, the rates of CKD were similar among the three groups. The incidence of anemia was much greater in the patients with cancer, both active and inactive, than in those without cancer (43.6%, 43.7%, and 26.7%, respectively; *p* < 0.001). The CAS group were less likely to be using antihypertensive medication and statins than those in the other groups (53.8%, 67.7%, and 55.9%; *p* = 0.002; and 10.2%, 17.1%, and 22.6%, respectively; *p* = 0.030)*.* We did not discover any significant difference in the number of patients receiving acute reperfusion therapy, such as intravenous thrombolysis and endovascular therapy (EVT), between the three groups. Table 1 summarizes the baseline characteristics.

**Table 1:** Characteristics of patients with stroke with and without cancer.

	No cancer (*N* = 1566)	Inactive Cancer (*N* = 263)	Cancer associated stroke (*N* = 39)	*p*-value
Age, mean ± SD, years	72.3 ± 14.34	73.4 ± 13.53	68.3 ± 16.04	*0.098*
Male, n (%)	62.1%	62.7%	48.7%	*0.225*
BMI, mean ± SD	24.63 ± 4.18	23.87 ± 4.30	22.67 ± 4.15	*0.001*^*⁎*^

Comorbidity				
Hypertension, %	90.9%	85.2%	66.7%	*0.001*^*⁎*^
Diabetes mellitus, %	45.2%	43.4%	33.3%	*0.290*
Old CVA, %	20.4%	16.0%	15.4%	*0.160*
End stage renal disease, %	4.7%	1.5%	2.6%	*0.006*^*⁎*^
Chronic kidney disease, %	42.6%	41.8%	25.6%	*0.086*
Peripheral artery disease, %	17.2%	7.6%	25.6%	*0.290*
Dyslipidemia, %	75.1%	66.5%	46.2%	*<0.001*^*⁎*^
Coronary artery disease, %	18.5%	17.9%	7.7%	*0.056*
Congestive heart failure, %	59.4%	34.2%	25.6%	*0.086*
Atrial fibrillation, %	19.1%	22.1%	7.7%	*0.019*^*⁎*^
Anemia, %	26.7%	43.7%	43.6%	*<0.001*^*⁎*^

Pre-stroke medication				
Anti-HTN medication, %	55.9%	67.7%	53.8%	*0.002*^*⁎*^
Anti-DM medication, %	24.0%	22.4%	15.4%	*0.406*
Anticoagulants, %	7.7%	9.1%	15.4%	*0.178*
Anti-platelet agents, %	24.5%	28.1%	25.6%	*0.442*
Statin, %	22.6%	17.1%	10.2%	*0.030*^*⁎*^

Stroke Severity				
NIHSS score, mean ± SD	7.2 ± 7.63	7.8 ± 8.06	9.3 ± 8.87	*0.135*

Initial vital signs				
SBP, mean ± SD	155.3 ± 29.6	149.6 ± 29.6	137.1 ± 29.9	*<0.001*^*⁎*^
DBP, mean ± SD	84.6 ± 18.4	81.76 ± 16.1	77.9 ± 13.4	*0.002*^*⁎*^
MAP, mean ± SD	108.0 ± 20.4	104.4 ± 18.7	97.7 ± 15.3	*<0.001*^*⁎*^
BT, mean ± SD	36.18 ± 0.71	36.25 ± 0.76	36.38 ± 0.77	*0.232*
HR, mean ± SD	79.6 ± 17.50	84.8 ± 19.09	86.9 ± 14.89	*<0.001*^*⁎*^
RR, mean ± SD	18.7 ± 3.94	19.1 ± 4.13	18.8 ± 3.72	*0.281*

Treatment of acute stroke				
IV-rtPA	6.5%	3.8%	0.3%	*0.094*
EVT	6.4%	4.2%	2.6%	*0.129*

Laboratory data revealed significantly lower levels of hemoglobin (11.2 g/dL, 12.5 g/dL, and 13.5 g/dL; *p* < 0.001), albumin (3.3 g/dL, 3.5 g/dL, and 3.6 g/dL; *p* = 0.009), and HbA1c (6.19%, 6.26%, and 6.57%; *p* = 0.005) in the patients with CAS than in those with inactive cancer and the control patients, respectively. The CAS group also had higher values of prothrombin time with the result converted into the international normalized ratio (PT/INR; 1.19, 1.15, and 1.08; *p* < 0.001) and C-reactive protein (CRP; 5.79 mg/L, 3.92 mg/L, and 0.06 mg/L; *p* < 0.001). Other laboratory data indicated no significant differences between the groups. Table 2 summarizes the results of laboratory examinations.

**Table 2:** Laboratory data of stroke patients with and without cancer.^a^

	No cancer (N = 1566)	Inactive cancer (N = 263)	Cancer associated stroke (N = 39)	P-value
*Median Hb (IQR)*	13.7 (2.6)	12.5 (3.15)	11.14 (3.95)	<0.001*
*Median INR (IQR)*	1.05 (0.11)	1.07 (0.18)	1.13 (0.265)	<0.001*
*Median Albumin (IQR)*	3.6 (0.7)	3.5 (0.85)	3.3 (0.85)	0.009*
*Median Creatinine (IQR)*	1.03 (0.49)	1.04 (0.505)	0.84 (0.5125)	*0.137*
*Median CRP (IQR)*	0.36 (1.3025)	0.98 (5.2125)	3.35 (8.495)	<0.001*
*Median HbA1c (IQR)*	6.0 (1.3)	5.9 (1.1)	5.6 (0.575)	0.019*
*Median Homocysteine (IQR)*	10.46 (5.56)	9.01 (4.7375)	8.42 (9.13)	*0.796*
*Median Fibrinogen (IQR)*	383.25 (141.9)	307.55 (280.45)	335.9 (325.32)	0.007*
*Median T-Chol. (IQR)*	161.0 (54)	157.0 (53)	161.0 (73)	*0.692*
*Median LDL (IQR)*	100.0 (48)	96.0 (43.55)	105.0 (49.5)	*0.351*

a Bold indicated significant difference between the 3 groups.

### 3.2 Prevalence of cancer types

**Fig. 2:**
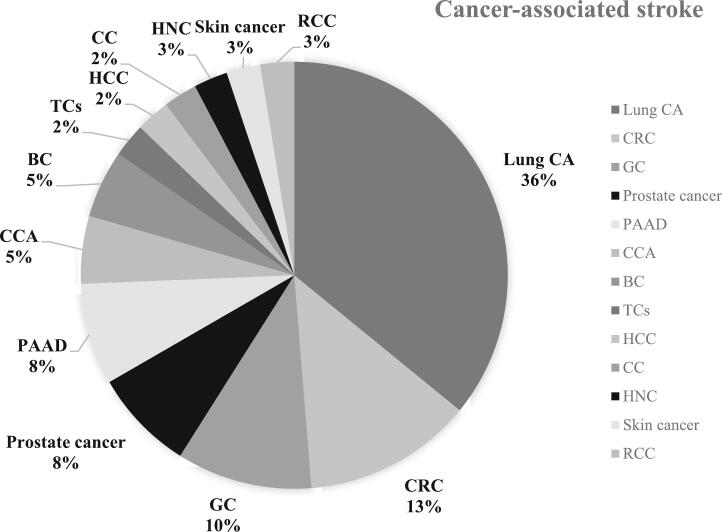
Distribution of cancer types in patients with CAS. Abbreviation: CA, cancer; CRC, colorectal cancer; GC, gastric cancer; PAAD, pancreatic adenocarcinoma; CCA, cholangiocarcinoma; BC, breast cancer; TC, thymic cancer; HCC, hepatocellular carcinoma; CC, cervical cancer; HNC, head and neck cancer; RCC, renal cell carcinoma.

Of the 39 patients with CAS, 18 (46.2%) were male. Lung cancer was the most common diagnosis (*n* = 14; 35.9%), followed by colorectal cancer (CRC; *n* = 5; 12.8%) and gastric cancer (*n* = 4; 10.3%). Three patients each had diagnoses of pancreatic and prostate cancer (*n* = 3; 7.7%), and two patients each had cholangiocarcinoma and breast cancer (*n* = 2; 5.1%). The group included one case each of thymic carcinoma, head and neck cancer (HNC), skin cancer, hepatocellular carcinoma (HCC), cervical cancer, and renal cell carcinoma. The data indicated differences in cancer type by sex; all patients with gastric cancer were male, and all patients with pancreas cancer were female. Fig. 2 summarizes the cancer type distribution in the CAS group.

**Fig. 3:**
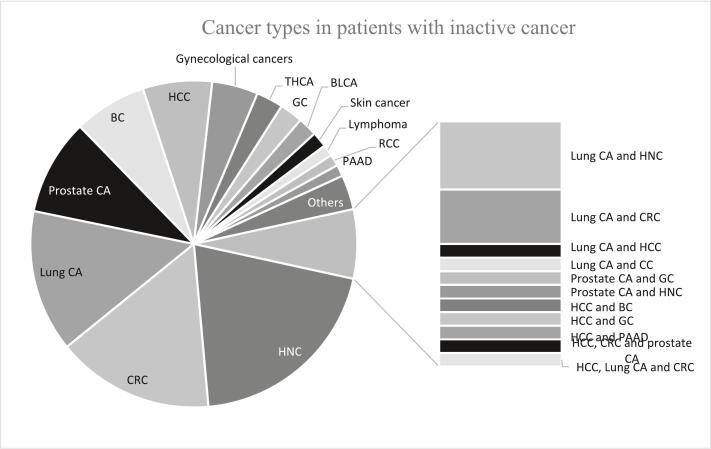
Distribution of cancer types in patients with inactive cancer. Abbreviations: CRC, colorectal cancer; HNC, head and neck cancer; CA, cancer; BC, breast cancer; HCC, hepatocellular carcinoma; THCA, thyroid cancer; GC, gastric cancer; BLCA, bladder cancer; RCC, renal cell carcinoma; PAAD, pancreatic adenocarcinoma.

By contrast, 166 (63.1%) of the patients with inactive cancer were male. Twenty-two patients had multiple cancers, 15 of whom were men. In the patients with multiple cancers, the three most common cancers were lung cancer (*n* = 12), HNC (*n* = 6), and HCC (n = 6). In the patients with a single type of cancer, the four most common cancers were HNC (*n* = 53; 20.2%), CRC (*n* = 41; 15.6%), lung cancer (*n* = 37; 14.1%), and prostate cancer (*n* = 25; 9.6%). However, the data indicated a sex-specific distribution of cancer types. In the male patients with a single type of cancer, the four most common cancers were HNC (*n* = 44; 29.1%), prostate cancer (n = 25; 16.6%), lung cancer (*n* = 23; 15.2%), and CRC (*n* = 22; 14.6%). Breast cancer ranked first in the female patients (*n* = 19; 21.1%), followed by lung cancer (*n* = 16; 17.8%) and CRC (*n* = 14; 15.6%). Female gynecological cancer ranked fourth when combining cervical, ovarian, uterine, and vulvar cancers (*n* = 12; 13.3%). Fig. 3 summarizes the distribution of cancer types in the patients with inactive cancer.

### 3.3 Outcome analysis

Table 3 details the mortality rate, stroke recurrence rate, and rate of good functional outcomes in the three groups. Throughout the observational period, the mortality rate was highest (*p* < 0.001) and the rate of good functional outcomes was lowest (*p* < 0.001) in the CAS group. Unexpectedly, during the 1-year follow-up, the stroke recurrence rate was highest in the group with inactive cancer (*p* = 0.045).

**Table 3:** Outcomes of patients with CAS, inactive cancer, or no cancer.

	No cancer (N = 1566)	Inactive Cancer (N = 263)	Cancer associated stroke (N = 39)	p-value
30-day mortality	74 (4.7%)	33 (12.5%)	7 (17.9%)	<0.001*
90-day mortality	120 (7.6%)	60 (22.8%)	14 (35.9%)	<0.001*
180-day mortality	160 (10.2%)	77 (29.3%)	17 (43.6%)	<0.001*
1-year mortality^a^	194 (12.4%)	89 (33.8%)	20 (51.3%)	<0.001*
1-year stroke recurrence	28 (1.8%)	11 (4.2%)	1 (2.6%)	0.045*
1-year functional independence(mRS≦2)	851 (54.3%)	97 (36.9%)	6 (15.4%_	<0.001*

a Bold indicated there is significant difference between the groups

#### 3.3.1 Cancer-associated stroke

Age, sex, and BMI did not affect the mortality rate in the CAS group. However, the mortality rate was higher in the patients with anemia than in those without at the 1-year follow-up, but the difference was not significant. Higher severity of stroke was associated with a higher odds ratio for 30-day mortality in both the univariate and multivariable analyses. However, the effect of stroke severity was weaker for 1-year mortality. Instead, baseline condition exerted a strong effect on the mortality rate. Coagulopathy, (e.g., elevated PT/INR) was also associated with a higher risk of 30-day mortality. A higher CRP level was consistently correlated with a higher mortality risk. Table 4 summarizes the results.

**Table 4:** Logistic regression models to predict mortality in CAS.

	Cancer-associated stroke							
	30-days mortality				1-year mortality			
	Crude OR		Multivariate OR		Crude OR		Multivariate OR	
Age	1.013 (0.960–1.069)	*0.645*			1.012 (0.972–1.054)	*0.559*		
Male	0.850 (0.163–4.426)	*0.847*			0.600 (0.169–2.135)	*0.430*		
Normal weight	Ref.							
Underweight	0.868 (0.125–6.026)	*0.886*			0.433 (0.106–1.761)	*0.242*		
Overweight and obesity	2.750 (0.162–46.792)	*0.484*			1.250 (0.089–17.653)	*0.869*		
DM	0.667 (0.111–3.990)	*0.657*			0.370 (0.096–1.435)	*0.151*		
HTN	0.606 (0.114–3.230)	*0.557*			1.361 (0.358–5.175)	*0.651*		
DL	0.147 (0.016–1.365)	*0.092*			0.600 (0.169–2.135)	*0.430*		
Normal renal function	Ref.							
CKD	3.125 (0.547–17.841)	*0.200*			4.308 (0.761–24.384)	*0.099*		
ESRD	0.000 (0.000-∞)	*1.000*			*0.000* (0.000-∞)	*1.000*		
Anemia	2.344 (0.393–13.964)	*0.350*			3.667 (0.954–14.092)	*0.059*		
CAD	1.611 (0.142–18.262)	*0.700*			0.281 (0.027–2.970)	*0.291*		
Afib	5.167 (0.282–94.501)	*0.268*			0.947 (0.055–16.309)	*0.970*		
Old stroke	1.167 (0.110–12.381)	*0.898*	0.593 (0.088–4.009)	*0.592*	1.069 (0.980–1.167)	*0.133*		
Initial NIHSS	1.163 (1.046–1.294)	0.005*	1.160 (1.011–1.332)	0.035*	1.069 (0.980–1.167)	*0.133*		
Initial SBP	0.999 (0.971–1.026)	*0.919*			0.994 (0.972–1.016)	*0.581*		
Hb	0.753 (0.542–1.046)	*0.091*			0.713 (0.527–0.965)	0.028*	0.661 (0.410–1.066)	a*0.089*
PT(INR)	116,332.8	0.007*	108,317.295	0.027*	894.013	0.011*	1.580	*0.915*
	(25.923–5.2E8)		(3.737–3.140E9)		(4.699–1.7E4)		(0.000–6851.161)	
Albumin	0.229 (0.039–1.339)	*0.102*			0.232 (0.051–1.052)	*0.058*		
Creatinine	0.920 (0.320–2.645)	*0.877*			0.560 (0.160–1.963)	*0.365*		
CRP	1.133 (0.998–1.285)	*0.053*			1.581 (1.091–2.290)	0.015*	1.094 (1.067–1.123)	0.016*
HbA1c	0.715 (0.258–1.983)	*0.519*			0.690 (0.386–1.236)	*0.212*		
HCY	0.908 (0.522–1.582)	*0.734*			0.908 (0.522–1.582)	*0.734*		
Fibrinogen	0.996 (0.988–1.004)	*0.345*			0.996 (0.99–1.004)	*0.334*		
Total Cholesterol	0.985 (0.956–1.016)	*0.335*			1.005 (0.990–1.021)	*0.515*		
LDL	0.950 (0.887–1.018)	*0.148*			1.004 (0.986–1.023)	*0.676*		

a Bold indicated there is significant difference between the groups

#### 3.3.2 Stroke with concurrent inactive cancer

Similar to the findings for the CAS group, the results for the group with inactive cancer indicated that the severity of stroke and coagulopathy (e.g., elevated PT/INR) were associated with 30-day mortality in both the univariate and multivariable analyses. Some vascular risk factors such as HTN and DL served as protective factors, whereas anemia constituted a risk factor for mortality in the univariate analysis but not in the multivariable analysis. Unexpectedly, coronary artery disease served as a protective factor for short-term mortality in the univariate analysis. However, the effect weakened with further analysis (Table 5).

**Table 5:** Logistic regression models to predict mortality in patients with stroke with inactive cancer.

	Inactive Cancer							
	30-days mortality				1-year mortality			
	Crude OR		Multivariate OR		Crude OR		Multivariate OR	
Age	0.983 (0.957–1.009)	*0.195*			0.989 (0.970–1.007)	*0.230*		
Male	0.502 (0.241–1.046)	*0.066*			0.641 (0.380–1.083)	*0.096*		
Normal weight	Ref.							
Underweight	0.839 (0.224–3.144)	*0.795*			4.857 (1.785–13.214)	0.002*	19.703	0.005*
							(2.492–155.762)	
Overweight and obesity	0.593 (0.262–1.340)	*0.209*			0.384 (0.212–0.696)	0.002*	0.667 (0.226–1.965)	*0.462*
DM	0.617 (0.286–1.330)	*0.218*			0.630 (0.372–1.065)	*0.085*		
HTN	0.140 (0.062–0.313)	<0.001*	0.333 (0.082–1.348)	*0.123*	0.330 (0.165–0.660)	0.002*	0.551 (0.111–2.741)	*0.466*
DL	0.206 (0.094–0.447)	<0.001*	0.574 (0.154–2.145)	*0.409*	0.414 (0.243–0.706)	0.001*	1.604 (0.515–a4.992)	*0.415*
Normal renal function	Ref.							
CKD	0.756 (0.346–0.1652)	*0.756*			0.963 (0.569–1.629)	*0.887*		
ESRD	6.600 (0.879–49.534)	*0.067*			5.941 (0.603–58.556)	*0.127*		
Anemia	5.054 (2.107–12.122)	<0.001*	0.405 (0.042–3.908)	*0.435*	4.946 (0.282–8.651)	<0.001*	1.291 (0.226–7.379)	*0.774*
CAD	0.118 (0.016–0.889)	0.038*	0.000 (0-∞)	*0.098*	0.577 (0.284–1.172)	*0.128*		
Afib	1.182 (0.502–2.783)	*0.702*			1.733 (0.951–3.161)	*0.073*		
Old stroke	0.505 (0.147–1.740)	*0.279*			0.677 (0.322–1.424)	*0.304*		
Initial NIHSS	1.104 (1.060–1.150)	<0.001*	1.086 (1.017–1.160)	0.014*	1.122 (1.081–1.165)	<0.001*	1.079 (1.015–1.148)	*0.015*
Initial SBP	0.983 (0.969–0.996)	0.011*	0.988 (0.963–1.014)	*0.362*	0.986 (0.976–0.995)	0.002*	0.987 (0.967–1.007)	*0.201*
Initial HR	1.036 (1.017–1.056)	<0.001*	1.027 (0.999–1.055)	*0.059*	1.043 (1.027–1.059)	<0.001*	1.065 (1.033–1.097)	<0.001*
Hb	0.659 (0.549–0.791)	<0.001*	0.848 (0.535–1.343)	*0.482*	0.644 (0.559–0.742)	<0.001*	0.887 (0.592–1.329)	*0.560*
PT(INR)	104.804	<0.001*	25.992	0.013*	61.878	<0.001*	2.111	*0.515*
	(15.579–705.042)		(1.988–339.756)		(10.642–359.775)		(0.222–20.045)	
Alb	0.409 (0.199–0.842)	0.015*	0.728 (0.240–2.211)	*0.575*	0.220 (0.115–0.422)	<0.001*	0.728 (0.240–2.208)	*0.575*
Crea	1.182 (0.923–1.515)	*0.185*			1.170 (0.907–1.508)	*0.226*		
CRP	1.070 (1.020–1.123)	0.006*	0.943 (0.863–1.029)	*0.188*	1.196 (1.113–1.284)	<0.001*	1.042 (0.959–1.133)	*0.330*
HbA1c	0.726 (0.448–1.178)	*0.195*			0.962 (0.763–1.213)	*0.743*		
HCY	1.032 (0.755–1.409)	*0.845*			1.067 (0.916–1.243)	*0.403*		
Fibrinogen	0.995 (0.990–1.000)	*0.053*			0.998 (0.995–1.002)	*0.426*		
Total Cholesterol	0.997 (0.988–1.007)	*0.572*			0.999 (0.995–1.004)	*0.770*		
LDL	0.997 (0.985–1.008)	*0.575*			0.996 (0.989–1.003)	*0.265*		

a Bold indicated there is significant difference between groups

The risk and protective factors changed during the 1-year follow-up period. Contrary to general expectations, the 1-year mortality rate was lowest in the patients with overweight or obesity; those who were underweight had a 19-fold greater risk of mortality than the patients with normal weight. Furthermore, we noted that increased heart rate correlated with a higher 1-year mortality risk even after adjustment.

The cumulative incidence of recurrent stroke (either hemorrhagic or ischemic) was higher in the CAS group than in the inactive cancer and control groups (5.1%, 4.6%, and 1.7%, respectively; *p* = 0.005). Unsurprisingly, the patients with CAS also exhibited the poorest functional outcomes, with only 15.6% of these patients having reached functional independence (mRS score ≤ 2) at the 1-year follow-up.

## 4 Discussion

Cancer and stroke have a complex relationship. Studies have focused either on patients with cancer or on patients with stroke and concomitant cancer. To the best of our knowledge, this study is the first to divide patients into diagnostic categories of CAS, inactive cancer, and noncancer. We believe that our findings elucidate the similarities and differences between these groups.

In the TVGHSR, 16.2% of patients had one or more cancer diagnoses before their acute stroke. This finding agrees with those of previous observational studies [6,19]. Lung cancer, CRC, and pancreatic cancer were most mentioned due to their association with elevated thromboembolism risk [6,[20], [21], [22]]. In addition, breast and prostate cancer were common in female and male patients, respectively. Our study noted that head and neck cancer was the most prevalent cancer type, encompassing 18% of the patients with stroke. However, these patients were primarily classified as patients with inactive cancer rather than CAS.

Studies have reported various CAS prevalence rates, which have ranged from as low as 2.3% [13] in a Turkish study and 5.2% in a Hong Kong study [12] to as high as 18.8% in a Japanese study [23]. Similar to that in the Turkish study, our study indicated CAS prevalence of 2.1% [13]. Also similar to previous studies, our study discovered that patients with CAS had fewer conventional vascular risk factors such as DL, HTN, and Afib. We also discovered a much lower incidence of ESRD but a higher rate of anemia in the CAS group. However, the prevalence of CKD was the same.

Researchers have proposed several mechanisms regarding the pathophysiology of CAS [24]. First, patients with CAS may have predisposing risk factors such as HTN, DM, DL, and Afib. Tumor-related etiologies include direct tumor effects—such as vessel compression, tumor emboli, and vessel invasion-induced arterial, venous, or sinovenous thrombosis—and cancer-associated coagulopathy, such as marantic endocarditis, thrombocytopenia, mucinous carcinoma, and disseminated intravascular coagulation. Treatment-related factors include chemotherapy-induced neurotoxicity, radiotherapy-induced vasculopathy, and treatment-related hematopoietic and perioperative complications such as transient hypoperfusion.

Our study identified the five most common cancer types in the CAS group as being lung cancer, CRC, gastric cancer, prostate cancer, and pancreatic adenocarcinoma. By contrast, the Taiwan Cancer Registry data indicate that the highest prevalences are for female breast, CRC, lung, and prostate cancers [25]. Gastrointestinal cancer ranked first in the patients with CAS when combining cholangiocarcinoma, CRC, gastric, and pancreatic cancers. Although the Registry database did not contain tissue type results, we assumed adenocarcinoma to be the most prevalent cancer type in the CAS group. Our findings slightly differed from those of an American study, in which patients with prostate cancer had a lower stroke risk than did control without cancer [26]. A Japanese study conducted by Gon et al. [27] found that lung, pancreatic, and CRC, particularly adenocarcinomas, were the types of cancer most frequently associated with ischemic stroke. Similar to a Hong Kong study [14], our study enrolled patients belonging to the southern Mongolian–Han population; however, the Hong Kong study reported that the most prevalent cancers in patients with stroke were breast cancer (17%), CRC (14%), nasopharyngeal cancer (13%), lung cancer (10%), and prostate cancer (8%) [14]. This finding implies that factors beyond genetics strongly influence the pathogenesis of CAS and may include environmental factors.

High rates of CRC and lung cancer persist in both Eastern and Western nations, but studies have reported various rates of nasopharyngeal, prostate, and breast cancers. Epidemiological differences may explain these discrepancies. Physicians should be aware of these differences when treating patients with suspected CAS.

In addition, we found no statistical differences in the rate of patients receiving acute reperfusion therapy such as intravenous recombinant tissue plasminogen activator or EVT between those with versus without cancer; however, a previous study noted a much lower rate in patients with cancer than in a control group [28]. Thus, our study excluded the effect of revascularization therapy on patient outcomes. Nevertheless, the 1-year mortality rate was significantly higher in the patients with CAS than in the patients with inactive cancer and in the non-cancer group. Thus, the poor outcomes of the patients with CAS may have been partly attributable to underlying malignancy rather than the type of acute stroke management.

This observational study determined that cancer negatively affected patient survival and functional outcomes in both the acute and chronic phases of stroke. In the patients with CAS, stroke severity was the most critical factor affecting the likelihood of short-term mortality. One study suggested that D-dimer level can act as a predictor of poor prognoses [29]. Although this study did not test D-dimer levels, we note that a higher PT/INR level may be an alternative biomarker of high mortality risk in both the short and long term. Studies have demonstrated that high-sensitivity CRP level may predict poor long-term functional outcomes and mortality [30]. In addition, we found that higher CRP levels predicted a higher rate of 1-year mortality in the CAS group.

Conventional vascular risk factors such as DL and HTN are known to have negative effects on stroke outcome. However, in our retrospective study, patients with inactive cancer and concurrent metabolic syndrome with DM and DL had better short-term and long-term outcomes. Both DM and DL acted as protective factors even after adjustment for other comorbidities. We also observed that a higher BMI lowered the risk of 1-year mortality in these patients. We postulated the following explanations for these conflicting findings. DM, high BMI, and DL all represent an “adequately-nourished state.” Patients with cancer have an increased risk of malnutrition and cancer cachexia, both of which may contribute to a higher mortality rate and worse functional outcomes. Consequently, the mortality rate may be lower in patients in the “adequately-nourished state.” Further prospective studies are required to verify this postulation.

## 5 Limitations

This study has several limitations. First, we conducted a single-center registry study in a tertiary referral hospital located in a well-developed urban area, which may have caused selection bias. The data may not reflect the proportions of stroke and concomitant cancer in the general population. Further research with a more detailed experimental design is warranted to investigate the epidemiology, pathophysiology, and outcomes of patients with stroke and concomitant cancer.

Because this study used depersonalized registry data, we could not identify specific cancer pathologies. Thus, we could not accurately classify patients by pathological type. We also lacked the staging and detailed treatment plans for each patient and thus could not reliably evaluate whether the patients were in the early, late, or recurrent phase, which would correspond to differing stroke risks. However, one study reported that the development of cancer up to 5 years before a stroke served as an independent risk factor for mortality and poorer outcomes [31]. Moreover, as a routine risk factor survey, our retrospective data did not include tests for the fibrin degradation product or D-dimer. Therefore, we cannot elaborate on the associations between those biomarkers and cancer and stroke outcomes.

## 6 Conclusion

Cancer constitutes a crucial etiology of cryptogenic stroke. To the best of our knowledge, this is the first report to analyze the differences between patients with CAS, patients with stroke and inactive cancer, and patients with stroke without cancer. Whereas lung and CRC cancers are the leading causes of cancer-related stroke worldwide, prostate and breast cancers are also prevalent in patients with stroke in the Southeast Asian population. HTN and DL appear less harmful in patients with cancer than in patients with stroke without cancer. Further studies are required to evaluate the different stroke etiologies in patients with active cancers and to devise optimal treatment strategies for such patients.

## Source of funding

This research received no external funding.

## Data sharing and data accessibility

Data sharing and data accessibility: All data were available in the corresponding author by reasonable request.

## CRediT authorship contribution statement

**Kang-Po Lee:** Writing – original draft, Conceptualization. **Hui-Chi Huang:** Formal analysis, Data curation. **Jui-Yao Tsai:** Resources, Data curation. **Li-Chi Hsu:** Writing – review & editing, Methodology.

## Declaration of Competing Interest

None.

## Appendix A Supplementary data

Supplementary 1: Logistic regression models to predict mortality in cancer-associated stroke; Supplementary 2: Logistic regression models to predict mortality in patients with stroke with inactive cancer Image 1
