# Supplementary material for: Effects of cancer on stroke recurrence and mortality: A single-center retrospective cohort study
Source: eNeurologicalSci. 2023 Jul 17;32:100474. doi: 10.1016/j.ensci.2023.100474 (PMC10374457; doi:10.1016/j.ensci.2023.100474)
Supplement: Supplementary file 1 — Supplementary 1: Logistic regression models to predict mortality in cancer-associated stroke; Supplementary 2: Logistic regression models to predict mortality in patients with stroke with inactive cancer [file mmc1.docx]

Supplementary table 1. Logistic regression models to predict mortality in CAS

|  | Cancer-associated stroke | | | | | | | | | | | | |
| --- | --- | --- | --- | --- | --- | --- | --- | --- | --- | --- | --- | --- | --- |
|  | 30-days mortality | | | | | | | 1-year mortality | | | | | |
|  | Crude OR | | | | | Multivariate OR | | Crude OR | | Multivariate OR | | | |
| Age | 1.013  (0.960-1.069) | | | *0.645* | |  |  | 1.012  (0.972-1.054) | *0.559* |  | |  | |
| Male | 0.850  (0.163-4.426) | | | *0.847* | |  |  | 0.600  (0.169-2.135) | *0.430* |  | |  | |
| Normal weight | | Ref. | | | | | | | | | | | |
| Underweight | 0.868  (0.125-6.026) | | | *0.886* | |  |  | 0.433  (0.106-1.761) | *0.242* |  | |  | |
| Overweight  and obesity | 2.750  (0.162-46.792) | | | *0.484* | |  |  | 1.250  (0.089-17.653) | *0.869* |  | |  | |
| DM | 0.667  (0.111-3.990) | | | *0.657* | |  |  | 0.370  (0.096-1.435) | *0.151* |  | |  | |
| HTN | 0.606  (0.114-3.230) | | | *0.557* | |  |  | 1.361  (0.358-5.175) | *0.651* |  | |  | |
| DL | 0.147  (0.016-1.365) | | | *0.092* | |  |  | 0.600  (0.169-2.135) | *0.430* |  | |  | |
| Normal renal function | | | Ref. | | | | | | | | | | |
| CKD | 3.125  (0.547-17.841) | | | *0.200* | | |  | 4.308  (0.761-24.384) | *0.099* |  | |  | |
| ESRD | 0.000  (0.000-∞) | | | *1.000* | |  |  | *0.000*  (0.000-∞) | *1.000* |  | |  | |
| Anemia | 2.344  (0.393-13.964) | | | *0.350* | |  |  | 3.667  (0.954-14.092) | *0.059* |  | |  | |
| CAD | 1.611  (0.142-18.262) | | | *0.700* | |  |  | 0.281  (0.027-2.970) | *0.291* |  | |  | |
| Afib | 5.167  (0.282-94.501) | | | *0.268* | |  |  | 0.947  (0.055-16.309) | *0.970* |  | |  | |
| Old stroke | 1.167  (0.110-12.381) | | | | *0.898* | 0.593  (0.088-4.009) | *0.592* | 1.069  (0.980-1.167) | *0.133* |  | |  | |
| Initial  NIHSS | 1.163  (1.046-1.294) | | | ***0.005^*^*** | | 1.160  (1.011-1.332) | ***0.035^*^*** | 1.069  (0.980-1.167) | *0.133* |  | |  | |
| Initial  SBP | 0.999  (0.971-1.026) | | | *0.919* | |  |  | 0.994  (0.972-1.016) | *0.581* |  | |  | |
| Hb | 0.753  (0.542-1.046) | | | *0.091* | |  |  | 0.713  (0.527-0.965) | ***0.028^*^*** | | 0.661  (0.410-1.066) | | *0.089* |
| PT(INR) | 116332.8 | | | ***0.007^*^*** | | 108317.295 | ***0.027^*^*** | 894.013 | ***0.011^*^*** | 1.580 | | *0.915* | |
|  | (25.923-5.2E8) | | | | | (3.737-3.140E9) | | (4.699-1.7E4) | | (0.000-6851.161) | | | |
| Albumin | 0.229  (0.039-1.339) | | | *0.102* | |  |  | 0.232  (0.051-1.052) | *0.058* |  | |  | |
| Creatinine | 0.920  (0.320-2.645) | | | *0.877* | |  |  | 0.560  (0.160-1.963) | *0.365* |  | |  | |
| CRP | 1.133  (0.998-1.285) | | | *0.053* | |  |  | 1.581  (1.091-2.290) | ***0.015^*^*** | 1.094  (1.067-1.123) | | ***0.016^*^*** | |
| HbA1c | 0.715  (0.258-1.983) | | | *0.519* | |  |  | 0.690  (0.386-1.236) | *0.212* |  | |  | |
| HCY | 0.908  (0.522-1.582) | | | *0.734* | |  |  | 0.908  (0.522-1.582) | *0.734* |  | |  | |
| Fibrinogen | 0.996  (0.988-1.004) | | | *0.345* | |  |  | 0.996  (0.99-1.004) | *0.334* |  | |  | |
| Total  Cholesterol | 0.985  (0.956-1.016) | | | *0.335* | |  |  | 1.005  (0.990-1.021) | *0.515* |  | |  | |
| LDL | 0.950  (0.887-1.018) | | | *0.148* | |  |  | 1.004  (0.986-1.023) | *0.676* |  | |  | |

Supplementary table 2. Logistic regression models to predict mortality in patients with stroke with inactive cancer

|  | Inactive Cancer | | | | | | | | | | | | | | | |
| --- | --- | --- | --- | --- | --- | --- | --- | --- | --- | --- | --- | --- | --- | --- | --- | --- |
|  | 30-days mortality | | | | | | | 1-year mortality | | | | | | | | |
|  | Crude OR | | | | Multivariate OR | | | Crude OR | | | | Multivariate OR | | | | |
| Age | 0.983  (0.957-1.009) | | | *0.195* |  |  | | 0.989  (0.970-1.007) | | *0.230* | |  | | | | |
| Male | 0.502  (0.241-1.046) | | | *0.066* |  |  | | 0.641  (0.380-1.083) | | *0.096* | |  | | |  | |
| Normal weight | | Ref. | | | | | | | | | | | | | | |
| Underweight | 0.839  (0.224-3.144) | | | *0.795* |  |  | | 4.857  (1.785-13.214) | | ***0.002**** | | 19.703 | | | ***0.005**** | |
|  |  |  |  |  |  |  |  |  |  |  |  | (2.492-155.762) | | | | |
| Overweight  and obesity | 0.593  (0.262-1.340) | | | *0.209* |  |  | | 0.384  (0.212-0.696) | | ***0.002**** | | 0.667  (0.226-1.965) | | | | *0.462* |
| DM | 0.617  (0.286-1.330) | | | *0.218* |  |  | | 0.630  (0.372-1.065) | | *0.085* | |  | | |  | |
| HTN | 0.140  (0.062-0.313) | | | ***<0.001**** | 0.333  (0.082-1.348) | | *0.123* | 0.330  (0.165-0.660) | | ***0.002**** | | 0.551  (0.111-2.741) | | | | *0.466* |
| DL | 0.206  (0.094-0.447) | | | ***<0.001**** | 0.574  (0.154-2.145) | | *0.409* | 0.414  (0.243-0.706) | | ***0.001**** | | 1.604  (0.515-4.992) | | | | *0.415* |
| Normal renal function | | Ref. | | | | | | | | | | | | | | |
| CKD | 0.756  (0.346-.1652) | | | *0.756* |  |  | | 0.963  (0.569-1.629) | | *0.887* | |  | | |  | |
| ESRD | 6.600  (0.879-49.534) | | | *0.067* |  |  | | 5.941  (0.603-58.556) | | *0.127* | |  | | |  | |
| Anemia | 5.054  (2.107-12.122) | | | ***<0.001**** | 0.405  (0.042-3.908) | | *0.435* | 4.946  (0.282-8.651) | | ***<0.001**** | | | 1.291  (0.226-7.379) | | | *0.774* |
| CAD | 0.118  (0.016-0.889) | | | ***0.038**** | 0.000  (0-∞) | *0.098* | | 0.577  (0.284-1.172) | | *0.128* | |  | | |  | |
| Afib | 1.182  (0.502-2.783) | | | *0.702* |  |  | | 1.733  (0.951-3.161) | | *0.073* | |  | | |  | |
| Old stroke | 0.505  (0.147-1.740) | | | *0.279* |  |  | | 0.677  (0.322-1.424) | | *0.304* | |  | | |  | |
| Initial  NIHSS | 1.104  (1.060-1.150) | | | ***<0.001**** | 1.086  (1.017-1.160) | ***0.014**** | | 1.122  (1.081-1.165) | | ***<0.001**** | | | | 1.079  (1.015-1.148) | | *0.015* |
| Initial  SBP | 0.983  (0.969-0.996) | | | ***0.011**** | 0.988  (0.963-1.014) | | *0.362* | 0.986  (0.976-0.995) | | ***0.002**** | | 0.987  (0.967-1.007) | | | | *0.201* |
| Initial  HR | 1.036  (1.017-1.056) | | | ***<0.001**** | 1.027  (0.999-1.055) | | *0.059* | 1.043  (1.027-1.059) | ***<0.001**** | | 1.065  (1.033-1.097) | | | | | ***<0.001**** |
| Hb | 0.659  (0.549-0.791) | | | ***<0.001**** | 0.848  (0.535-1.343) | | *0.482* | 0.644  (0.559-0.742) | ***<0.001**** | | | 0.887  (0.592-1.329) | | | | *0.560* |
| PT(INR) | 104.804 | | ***<0.001**** | | 25.992 | ***0.013**** | | 61.878 | ***<0.001**** | | | 2.111 | | | *0.515* | |
|  | (15.579-705.042) | | | | (1.988-339.756) | | | (10.642-359.775) | | | | (0.222-20.045) | | | | |
| Alb | 0.409  (0.199-0.842) | | | ***0.015**** | 0.728  (0.240-2.211) | | *0.575* | 0.220  (0.115-0.422) | ***<0.001**** | | | | 0.728  (0.240-2.208) | | | *0.575* |
| Crea | 1.182  (0.923-1.515) | | | *0.185* |  |  | | 1.170  (0.907-1.508) | | *0.226* | |  | | |  | |
| CRP | 1.070  (1.020-1.123) | | | ***0.006**** | 0.943  (0.863-1.029) | | *0.188* | 1.196  (1.113-1.284) | ***<0.001**** | | | | 1.042  (0.959-1.133) | | | *0.330* |
| HbA1c | 0.726  (0.448-1.178) | | | *0.195* |  |  | | 0.962  (0.763-1.213) | | *0.743* | |  | | |  | |
| HCY | 1.032  (0.755-1.409) | | | *0.845* |  |  | | 1.067  (0.916-1.243) | | *0.403* | |  | | |  | |
| Fibrinogen | 0.995  (0.990-1.000) | | | *0.053* |  |  | | 0.998  (0.995-1.002) | | *0.426* | |  | | |  | |
| Total  Cholesterol | 0.997  (0.988-1.007) | | | *0.572* |  |  | | 0.999  (0.995-1.004) | | *0.770* | |  | | |  | |
| LDL | 0.997  (0.985-1.008) | | | *0.575* |  |  | | 0.996  (0.989-1.003) | | *0.265* | |  | | |  | |
